# An Integrative Roadmap for Advancing Colorectal Cancer Organoid

**DOI:** 10.3390/biomedicines14010248

**Published:** 2026-01-22

**Authors:** Youqing Zhu, Ke He, Zhi Shi

**Affiliations:** Cancer Minimally Invasive Therapies Centre, Guangdong Second Provincial General Hospital, Department of Cell Biology & Institute of Biomedicine, Guangdong Provincial Biotechnology & Engineering Technology Research Center, Guangdong Provincial Key Laboratory of Bioengineering Medicine, Guangdong Basic Research Center of Excellence for Natural Bioactive Molecules and Discovery of Innovative Drugs, Genomic Medicine Engineering Research Center of Ministry of Education, MOE Key Laboratory of Tumor Molecular Biology, National Engineering Research Center of Genetic Medicine, State Key Laboratory of Bioactive Molecules and Druggability Assessment, College of Life Science and Technology, Jinan University, Guangzhou 510632, China; jinanxuezi@stu2023.jnu.edu.cn (Y.Z.); heke8@mail3.sysu.edu.cn (K.H.)

**Keywords:** CRC organoid, multi-omics, organoid-immune co-culture, mini colon, drug screening, personalized treatment

## Abstract

Colorectal cancer (CRC) remains one of the leading causes of cancer-related mortality worldwide. Compared with traditional two-dimensional (2D) models, patient-derived CRC organoids more faithfully preserve the genomic, transcriptomic, and architectural features of primary tumors, making them a powerful intermediate platform bridging basic discovery and clinical translation. Over the past several years, organoid systems have rapidly expanded beyond conventional epithelial-only cultures toward increasingly complex architectures, including immune-organoid co-culture models and mini-colon systems that enable long-term, spatially resolved tracking of tumor evolution. These advanced platforms, combined with high-throughput technologies and clustered regularly interspaced short palindromic repeats (CRISPR)-based functional genomics, have substantially enhanced our ability to dissect CRC mechanisms, identify therapeutic vulnerabilities, and evaluate drug responses in a physiologically relevant context. However, current models still face critical limitations, such as the lack of systemic physiology (e.g., gut–liver or gut–brain axes), limited standardization across platforms, and the need for large-scale, prospective clinical validation. These gaps highlight an urgent need for next-generation platforms and computational frameworks. The development of high-throughput multi-omics, CRISPR-based perturbation, drug screening technologies, and artificial intelligence-driven predictive approaches will offer a promising avenue to address these challenges, accelerating mechanistic studies of CRC, enabling personalized therapy, and facilitating clinical translation. In this perspective, we propose a roadmap for CRC organoid research centered on two major technical pillars: advanced organoid platforms, including immune co-culture and mini-colon systems, and mechanistic investigations leveraging multi-omics and CRISPR-based functional genomics. We then discuss translational applications, such as high-throughput drug screening, and highlight emerging computational and translational strategies that may support future clinical validation and precision medicine.

## 1. Introduction

In recent years, colorectal cancer (CRC) has remained one of the most prevalent and lethal malignancies worldwide [1]. In the United States, an estimated 152,810 new CRC cases in 2024 include 106,590 colon cancers and 46,220 rectal cancers, with approximately 53,010 deaths projected [1]. These statistics underscore the substantial public health burden of CRC and highlight the urgent need for improved experimental models to study tumor biology and therapy response. Despite substantial advances in understanding the molecular and genetic landscape of CRC over the past decades, these discoveries have not yet translated into proportional clinical benefits for patients [2]. Similarly to other cancer types, a major contributing factor is the lack of experimental models that faithfully recapitulate in vivo conditions and reliably support disease modeling and drug screening [2,3]. Traditional two-dimensional (2D) cancer cell lines, while highly accessible and cost-effective, fail to preserve intratumoral heterogeneity, three-dimensional (3D) tissue architecture, and clinically relevant drug response patterns. Patient-derived xenograft (PDX) models partially retain tumor heterogeneity and histopathological features; however, their widespread application is constrained by low engraftment efficiency, prolonged establishment timelines, high maintenance costs, limited scalability, and the progressive replacement of the human tumor microenvironment by murine stromal components [4,5]. Genetically engineered mouse models (GEMMs) enable the investigation of tumor initiation and progression within an intact immune system, yet they often rely on predefined genetic alterations, exhibit long and labor-intensive breeding cycles, and incompletely capture the molecular diversity and evolutionary trajectories observed in human colorectal cancer [6]. These limitations collectively underscore the need for alternative experimental systems that combine human relevance, experimental flexibility, and scalability. This limitation has, in part, driven the increasing interest in and development of organoid technology. In contrast, patient-derived organoids (PDOs) which faithfully preserve the genomic, transcriptomic, and architectural characteristics of primary tumors have emerged as a powerful intermediate model bridging basic discovery and clinical translation, with applications spanning drug screening and disease modeling [7,8,9,10,11,12,13,14,15]. Compared with PDX and GEMM platforms, PDOs offer distinct advantages in terms of rapid establishment, cost-effectiveness, genetic manipulability, and suitability for high-throughput drug screening, while maintaining patient-specific tumor heterogeneity [4]. For example, PDOs established from surgical specimens can be assessed for consistency with both the histological features of the original tumors and the resulting organoids, followed by drug sensitivity testing. With the rapid advancement of high-throughput multi-omics technologies in recent years particularly immune-related co-culture systems, multi-omics analyses, and engineered platforms, the applications of CRC organoids in mechanistic studies and personalized medicine have greatly expanded.

**Figure 1:**
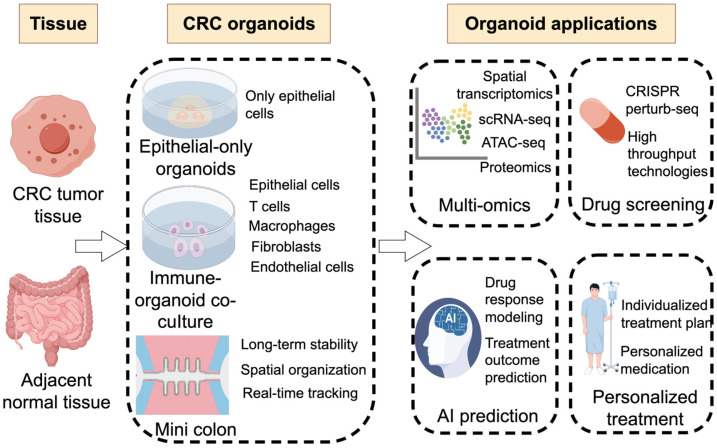
Overview of CRC organoid-based platforms and translational applications. AI, artificial intelligence. ATAC-seq, Assay for transposase-accessible chromatin with high throughput sequencing. CRC, colorectal cancer. CRISPR, clustered regularly interspaced short palindromic repeats. scRNA, single-cell RNA sequencing.

However, although PDOs offer distinct advantages, challenges in reproducibility and standardization of PDO culture remain. The field also lacks an integrated framework that unifies tumor evolution, immune–tumor interactions, and next-generation engineered organoid systems. Here, we outline future directions and propose an integrative roadmap to advance CRC organoid research, bridging mechanistic insights and clinical translation, as summarized in Figure 1 (drawn using the online platform Figdraw 2.0). This perspective focuses on representative advances, key challenges, and emerging directions, and does not aim to provide a comprehensive systematic review or meta-analysis.

## 2. Multi-Omics Integration Enables Mechanistic Insights from CRC Organoids

The integration of single-cell sequencing with various multi-omics approaches has provided powerful tools to dissect cellular heterogeneity, lineage dynamics, and tumor microenvironment (TME) interactions at unprecedented resolution. Recent advances in organoid technology, combined with these high-resolution approaches, have enabled researchers to investigate the cellular and molecular mechanisms driving CRC progression and metastasis with unprecedented detail. These models allow for the dissection of tumor-intrinsic programs, TME interactions, and lineage plasticity in a patient-specific context. Subsequently, several studies have employed organoid systems together with multi-omics techniques to reveal key insights into CRC biology.

Building on the principle that patient-derived CRC organoids faithfully recapitulate tumor features, several studies have leveraged organoid systems in combination with single-cell and multi-omics approaches to dissect tumor-intrinsic programs, lineage plasticity, and TME interactions at high resolution. Wang et al. established both tumor- and normal tissue-derived organoids from patient CRC tissues and their paired adjacent normal colon tissues, and performed single-cell RNA sequencing (scRNA-seq), whole-genome/exome sequencing, and DNA methylation analyses on both the original tissues and corresponding organoids [16]. They found that tumor-derived organoids faithfully recapitulate the transcriptomic, gene regulatory, and genomic features of the primary tumors, while normal-derived organoids maintain normal genomic and DNA methylation profiles but partially acquire tumor-like transcriptomic characteristics, including activation of certain cancer-associated genes [16]. Building on this foundation, Moorman et al. performed single-cell sequencing on matched primary CRC, metastatic CRC, and normal colon tissues, and subsequently employed matched organoid models to interrogate tumor-intrinsic plasticity and microenvironmental influences [17]. They revealed a continuous trajectory and validated the role of the transcription factor *PROX1* in restricting the atypical state. Furthermore, they linked this program to clinical outcomes, suggesting its potential as a biomarker and therapeutic target. Extending these analyses to gene-specific perturbations, Cammareri et al. generated *ATRX* knockout (KO) mouse CRC organoids via clustered regularly interspaced short palindromic repeats (CRISPR)/Cas9-mediated deletion of *ATRX*, and performed ATAC-seq, RNA sequencing, and scRNA-seq analyses [18]. They found that *ATRX* loss impairs the activity of the colonic lineage-determining transcription factor HNF4A, thereby promoting tumor invasion and metastasis. Functional validation in patient-derived *ATRX* KO human CRC organoids recapitulated similar phenotypic changes, supporting the findings from the mouse model. Finally, Li et al. systematically mapped carcinoma–TME interactions in human CRC tissues by performing scRNA-seq on primary tumors and adjacent normal colon tissues, combined with spatial proteomics and single-molecule fluorescence in situ hybridization to visualize key ligand/receptor expression and cell–cell spatial relationships [19]. They found that tumor epithelial cells engage in extensive receptor–ligand interactions with immune and stromal cells in vivo, which are largely lost when tumor cells are cultured as tumor-derived organoids. To functionally recapitulate these interactions, the authors performed co-culture experiments introducing human monocyte-derived macrophages, with or without cancer-associated fibroblasts, into tumor-derived organoids cultures, which induced immunosuppressive and pro-tumor gene expression programs. These findings provide a reductionist platform for modeling human carcinoma–TME interactions and highlight the importance of TME components in regulating tumor cell states [19].

Collectively, these studies demonstrate that single-cell and spatial datasets can illuminate intra-tumoral heterogeneity and spatial organization, revealing dynamic state transitions across diverse TME compartments, encompassing both immune and stromal populations, and suggest that these mechanistic insights from multi-omics analyses could ultimately inform clinical applications, such as the identification of predictive biomarkers or the stratification of patients for personalized therapy. However, current applications still primarily focus on descriptive characterization of cell-intrinsic genetic and transcriptional programs within CRC organoids, and the translation of such high-resolution insights into clinically actionable biomarkers remains limited. Bridging this gap will require integrative analytical frameworks capable of systematically linking multi-omics heterogeneity to treatment response trajectories across large, well-annotated patient cohorts.

Overall, single-cell and spatial multi-omics technologies have substantially expanded both the analytical resolution and mechanistic interpretability of CRC organoid systems. Yet, to date, these approaches have largely remained within the realm of biological discovery rather than serving as practical tools to guide clinical decision-making. The development of robust, reproducible, and clinically deployable biomarker models, anchored in multi-omics features and rigorously validated across independent cohorts and experimental platforms, represents a critical unmet need and will ultimately determine the translational impact of next-generation CRC organoid research. Although these approaches provide high-resolution mechanistic insights, translating them into functional platforms for modeling immunotherapy responses requires integrating organoids with their native immune components.

## 3. CRC Organoid-Immune Co-Culture Platforms for Modeling TME

CRC organoid models have been extensively studied and applied; however, traditional organoid systems predominantly preserve only the epithelial cancer cell compartment, whereas immune and stromal components of the TME are largely lost. This is primarily due to technical limitations in establishing long-term, stable co-cultures of multiple cell types, including fibroblasts, endothelial cells, and resident immune populations, under conditions optimized for epithelial cell expansion. To move from descriptive multi-omics studies to functional modeling, several research groups have developed CRC organoid-immune co-culture platforms, which partially retain tumor-associated immune or stromal cells and enable functional studies of immunotherapy responses.

Building on these advances, Neal et al. established patient-derived CRC organoids from primary tumor tissues while preserving the native TME using an air–liquid interface (ALI) system [20]. They performed scRNA-seq on dissociated PDOs, capturing both tumor epithelial cells and immune cells and supplemented this with V(D)J sequencing to profile the immune receptor repertoires of the lymphocytes. The PDOs retained the cellular composition, immune receptor diversity, and transcriptomic states of the original tumors, while functional assays demonstrated that anti–PD-1/PD-L1 treatment activated T cells and induced tumor cell killing [20]. Building on these PDO-immune co-culture systems, Esposito et al. established patient-derived CRC organoids from primary tumor tissues and constructed autologous immune-organoid co-culture platforms using patient-matched T cells and myeloid-derived suppressor cells to model immune checkpoint inhibitor (ICI) responses in vitro [21]. Through these co-culture systems, they identified a set of cancer-specific tissue markers (e.g., *REG4*, *MUC1*, *MUC5AC*) whose high expression in PDOs correlated with resistance to ICIs. Functional validation demonstrated that knocking out the key gene *REG4* in resistant PDOs restored sensitivity to T-cell–mediated tumor killing under anti–PD-1 treatment. This work provides a clinically relevant ex vivo platform to dissect mechanisms of ICI resistance and highlights potential biomarkers for patient stratification [21]. Further extending these approaches, Zhang et al. established autologous co-culture platforms using microsatellite stability (MSS) CRC patient-derived organoids and their matched tumor-infiltrating lymphocytes (TILs) [22]. They treated these co-cultures with the bacterial metabolite butyrate, either alone or in combination with anti-PD-1 antibodies, and found that butyrate significantly enhances the tumor-killing activity of TILs, with the strongest effect observed when combined with PD-1 blockade. The scRNA-seq analysis revealed that butyrate preferentially expands a cytotoxic GNLY^+^ CD8^+^ T cell subset, which expresses higher levels of cytotoxic and chemokine genes. Mechanistic studies further showed that butyrate activates NF-κB (p65) signaling, leading to upregulation of *GNLY* in CD8^+^ T cells and thereby boosting antitumor immunity. This study demonstrates a clinically relevant ex vivo microbiota-metabolite-immune-organoid platform and highlights butyrate, or its downstream pathways, as a potential adjuvant to improve the efficacy of ICIs in CRC [22].

Collectively, these studies illustrate that CRC organoid-immune co-culture platforms can preserve patient-specific tumor and immune components, recapitulate immunotherapy responses ex vivo, and provide powerful systems to dissect mechanisms of ICI resistance, holding strong promise for personalized immunotherapy testing. Notably, tumor-intrinsic factors, such as microsatellite instability (MIS) status, can influence immune activation in these platforms, with MIS-H CRCs generally exhibiting more robust responses than MSS CRCs. In practice, establishing these co-cultures entails technical challenges including matching patient-derived TILs to organoids, maintaining immune cell viability, and balancing conditions for both epithelial and immune compartments, but addressing these constraints is critical to maximize the translational potential of PDO-immune co-culture systems. In addition, an important limitation is that PDOs mostly capture short-term dynamics. To better model long-term spatial architecture and cellular interactions, engineered mini-colon systems have been developed.

## 4. Advances Toward Mini-Colon Systems

Building on the need to capture long-term dynamics and spatial architecture, Lutolf et al. developed highly physiologically relevant mini-colon organoid models that recapitulate colorectal tumorigenesis in vitro [23,24,25]. This model faithfully mimics CRC initiation and exhibits a human intestine-like cellular renewal capacity, diverse cell types and differentiation states, and the ability to be maintained long-term without passaging. Moreover, it enables real-time tracking at single-cell resolution over several weeks, allowing the analysis of interactions among distinct cellular subpopulations. Compared with conventional PDOs and ALI co-culture systems, mini-colon organoids offer greater structural complexity and long-term stability, as well as the ability to monitor dynamic processes such as clonal evolution and microenvironment remodeling. However, these advantages come at higher technical complexity and cost, and their scalability remains more limited than that of standard PDO cultures [23,24,25]. ALI systems, in contrast, allow retention of native immune components in shorter-term experiments but lack the long-term spatial organization of mini-colon models, while conventional PDOs are simpler, more cost-effective, and easily scalable but predominantly preserve the epithelial compartments and short-term dynamics [20]. This comparison underscores that mini-colon systems complement, rather than replace, PDO and ALI platforms.

The scRNA-seq confirmed that mini-colon organoids develop mature, functional cellular lineages and maintain structural stability over time. Upon blue-light-induced oncogene activation, malignant tumors arise at predefined locations within the organoids, which can be monitored at single-cell resolution for weeks without disrupting culture integrity [23]. Tumor cells generated in these organoids also form tumors in mice with comparable efficiency and pathology, demonstrating that the mini-colon system faithfully models colorectal tumorigenesis [23].

These mini-colon organoids reconstruct key architectural and functional features of the colorectal TME, enabling precise interrogation of immune-evasion mechanisms and pharmacological responses. Their flexibility, including compatibility with gene editing, live imaging, and high-throughput drug screening combined with the ability to capture patient-specific tumor heterogeneity, provides greater versatility than traditional animal models for dissecting disease mechanisms and identifying therapeutic vulnerabilities [24]. The transformative aspect of mini-colon systems lies in their integration of spatial organization, long-term stability, and real-time tracking [25]. This allows monitoring of early tumor initiation, clonal evolution, and microenvironment remodeling over several weeks [25]. However, despite recapitulating complex cellular processes previously accessible only in vivo, mini-colon organoids lack systemic physiological features, including drug metabolism, gut–liver and gut–brain communication, and distributed immune interactions, and thus cannot fully replace animal or whole-organism models.

Nevertheless, mini-colon systems represent a pivotal direction for next-generation engineered organoid technologies. Future improvements enabling scalable, cost-effective production and incorporation of vascular, neuronal, or microbiota components could enhance translational relevance, accelerate studies on tumor initiation, metastasis, and therapeutic response, and strengthen their role as a complementary platform in precision oncology and multi-tissue disease modeling. While mini-colon systems enable real-time tracking and functional interrogation, integrating gene editing approaches allow dissection of genetic determinants of tumor initiation, progression, and therapy response.

## 5. CRISPR-Based Functional Genomics in CRC Organoids

Traditional CRISPR screening has relied heavily on immortalized cell lines and in vitro models, which often fail to recapitulate the complex biology of CRC progression in vivo. In contrast, organoid models preserving tissue architecture, differentiation hierarchies, and patient-specific features provide an ideal and physiologically relevant platform for high-fidelity functional genomics studies. As such, to interrogate the genetic basis of CRC progression in physiologically relevant contexts, researchers have integrated CRISPR/Cas9-based gene editing with organoid systems. The feasibility of this strategy was first demonstrated by the Sato group, who applied CRISPR/Cas9 in human normal intestinal organoids to introduce key CRC driver mutations (e.g., *APC* and *KRAS*), thereby generating models that recapitulate the adenoma-carcinoma sequence [26]. This pioneering work established organoids not only as structural surrogates of native tissue but also as versatile platforms for functional investigation of tumor initiation [26]. Building on this foundation, Michels et al. established a pooled CRISPR/Cas9 screening platform in human colon organoids with pre-malignant backgrounds, enabling high-throughput functional testing in vitro and in vivo using xenografts [27]. This platform identified additional tumor suppressors and context-dependent fitness genes, highlighting the potential of organoid-based CRISPR screens to interrogate early tumorigenesis in a physiologically relevant setting [27]. Extending the application of organoid-based CRISPR screening beyond tumor initiation, Wang et al. performed a forward genetic screen in engineered CRC organoids and identified metastasis-specific regulatory genes, including *CTNNA1* and *BCL2L13* [28]. These findings demonstrated that organoid platforms can be leveraged not only to model early oncogenic transformation but also to elucidate gene networks governing metastatic progression [28]. Finally, Lin et al. performed an unbiased CRISPR knockout screen targeting the entire repertoire of transcription factor-encoding genes in adult small intestinal organoids, identifying ZNF800 as a master repressor that inhibits enteroendocrine cell differentiation, revealing new regulatory layers involving transcription factors such as *PAX4* and *NEUROG3*, and showcasing organoid-based CRISPR utility in dissecting cell-fate determination [29].

Collectively, these studies illustrate the growing power of organoid-based CRISPR screening. Compared with conventional 2D cell lines, organoids offer substantially greater physiological relevance by preserving tissue architecture, cellular hierarchies, and microenvironmental cues. Integrating CRISPR perturbation screens with immune co-culture CRC organoids or next-generation platforms such as mini-colons may enable comprehensive dissection of tumor evolution, immunotherapy resistance mechanisms, and pharmacological vulnerabilities. Successful implementation of these approaches requires careful consideration of editing efficiency, clonal representation, and quality control to ensure robust and interpretable screening outcomes. Coupling CRISPR perturbations with single-cell readouts, such as Perturb-seq or Perturb-ATAC, enables high-resolution mapping of gene regulatory circuits. Additionally, coupling CRISPR perturbations with functional assays and drug screens establishes a platform for mechanistic insights and precision therapeutic discovery. These approaches advance both mechanistic understanding and precision oncology applications.

## 6. Organoid-Based Platforms for High-Throughput Drug Screening

Beyond functional genomics, organoids also serve as scalable platforms for drug discovery, enabling genotype- and phenotype-guided therapeutic testing. Building on this premise, Toshimitsu et al. developed a scalable PDO screening pipeline (applied to 20 CRC-derived organoids and 6 normal colon organoids, screened against a 56-compounds panel) and integrated DNA-methylation and transcriptomic profiling to interpret drug responses [30]. This organoid screen identified epigenetic vulnerabilities most notably sensitivity to the BET inhibitor JQ1 and linked CIMP-associated *CHFR* silencing to paclitaxel sensitivity, illustrating how PDOs-based multi-omics can reveal genotype-dependent or epigenotype-dependent therapeutic opportunities [30]. However, these epithelial-only assays do not capture immune or stromal modulation of drug response. Extending the clinical-scale utility of PDOs, Mao et al. implemented a drug-repurposing pipeline testing 335 compounds on CRC PDOs and identified 34 candidate agents with anti-tumor activity. Hits were characterized by transcriptomic response signatures (e.g., differentiation induction, metabolic inhibition, cell-cycle arrest, immune-related programs), and several candidates were validated in immunodeficient xenografts [31]. These results underscore the power of PDO-based repurposing screens to rapidly nominate clinically actionable compounds, while also highlighting their limitation for predicting immune-mediated therapeutic effects. To scale organoid drug evaluation, recent advances in 3D bioprinting and extracellular matrix (ECM) engineering produce printable bioinks (including decellularized ECM-based composites) that support high post-printing viability and preserve organoid polarity and multicellular architecture [32]. Such printed organoid arrays are compatible with automated, high-throughput readouts and therefore facilitate systematic drug testing at larger scale.

However, similar to standard PDO assays, these platforms often lack immune and stromal components, and their scalability and cross-study comparability can also be influenced by organoid state parameters such as size, passage number, and viability. Addressing the remaining limitations in TME representation and clinical scalability will be critical for developing next-generation CRC organoid platforms.

## 7. Future Perspectives and Key Challenges

**Figure 2:**
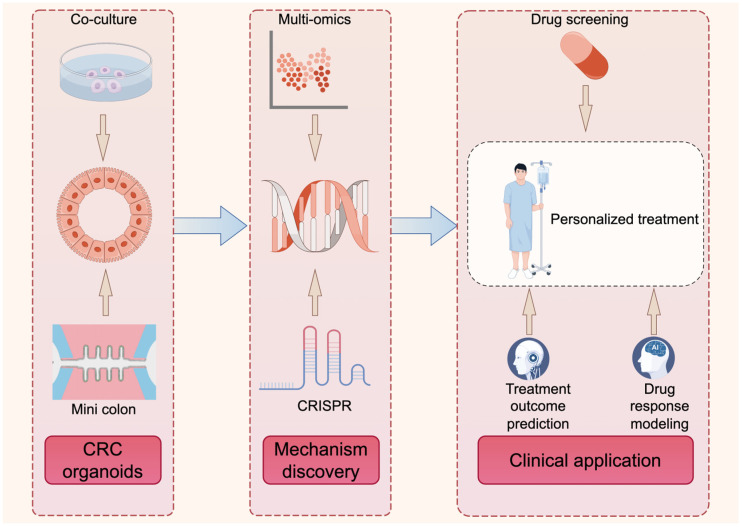
An integrative roadmap from CRC organoid modeling to clinical translation. CRC, colorectal cancer. CRISPR, clustered regularly interspaced short palindromic repeats.

Based on the trends discussed above, the future development of CRC organoid research is expected to move beyond descriptive modeling toward more integrated, functional, and predictive systems. Rather than serving solely as in vitro surrogates of patient tumors, next-generation CRC organoids are likely to become dynamic, modular platforms that connect mechanistic interrogation with therapeutic decision-making in a clinically meaningful way. These emerging directions can be integrated into a unified conceptual framework, as illustrated in Figure 2 (drawn using the online platform Figdraw 2.0).

We anticipate that future developments in CRC organoid research will advance along three major, interconnected directions. In terms of organoid model development, organoid systems will continue to evolve from conventional epithelial-only cultures toward increasingly complex architectures, including immune co-culture models, mini-colon systems, and, ultimately, fully integrated organoid platforms that incorporate key cellular components of the TME, such as immune cells, fibroblasts, endothelial cells, and ECM. These advanced systems hold promise not only for more faithful recapitulation of the in vivo tumor ecosystem, but also for large-scale screening and automation, potentially enabling the establishment of standardized organoid factories that can be embedded into clinical trials and translational research pipelines. From a mechanistic perspective, the combination of CRISPR-based gene perturbation technologies with multi-omics profiling and real-time imaging will allow systematic dissection of gene regulatory networks, lineage plasticity, and microenvironment-dependent signaling pathways. By introducing targeted or unbiased genetic perturbations into increasingly sophisticated organoid models, researchers will be able to identify early oncogenic drivers, modulators of immune evasion, and context-dependent vulnerabilities that are otherwise difficult to capture in conventional model systems. From a predictive perspective, advances in artificial intelligence (AI) and machine learning will enable the integration of baseline molecular features (such as genomic and transcriptomic profiles) with dynamic, longitudinal data derived from organoid responses to treatment (including single-cell sequencing and spatial omics). Conceptualizing treatment response as a dynamic trajectory rather than a static endpoint will allow the construction of predictive models capable of forecasting patient-specific sensitivity or resistance to various therapies, including chemotherapy, targeted agents, and ICI. In this way, CRC organoids may gradually evolve into personalized, data-driven platforms for therapeutic optimization. These three directions are interdependent, with advances in organoid models facilitating mechanistic studies, which in turn enhance AI-driven predictive capabilities.

To translate these conceptual advances into practice, medium-term goals may include integration of organoid platforms into early-phase clinical trials and development of standardized standard operating procedures for culture, co-culture, and drug testing. Short-term objectives could focus on harmonizing protocols across laboratories, improving reproducibility, and establishing shared data standards. These benchmarks will help guide coordinated progress toward fully realizing the potential of organoid-guided personalized medicine. Despite these promising directions, several critical challenges must be addressed before CRC organoids can be widely adopted in clinical practice. A major limitation lies in the lack of standardization, which is characterized by substantial inter-laboratory variability in matrices, culture conditions, growth factor formulations, and co-culture protocols, thereby compromising reproducibility and comparability across studies. In addition, the current workflow from patient sampling to organoid establishment, drug testing, and data interpretation remains time-consuming and costly, which restricts its feasibility for rapid clinical decision-making, particularly in time-sensitive settings such as neoadjuvant or metastatic disease management.

Furthermore, although recent advances in co-culture systems and chip-based platforms have improved the representation of the TME, fully reconstructing its complexity, including vascular networks, lymphatic structures, stromal heterogeneity, and microbiota remains an unresolved challenge. Finally, accumulating evidence demonstrates that organoid-based drug response profiles can serve as clinically relevant predictors of patient outcomes. In a large multicenter cohort of metastatic CRC patients, PDOs established from 205 biopsy samples accurately predicted treatment outcomes during systemic therapy, particularly for 5-fluorouracil and oxaliplatin, with strong associations with both progression-free and overall survival [33]. Similarly, in a prospective blinded study of stage IV CRC, PDO drug responses predicted individual chemotherapy efficacy with an overall accuracy of approximately 80%, supporting their potential as predictive biomarkers [34]. Collectively, these findings illustrate the utility of PDOs for clinical validation of therapeutic response. Despite these promising results, robust confirmation in large-scale, prospective, multicenter trials (including, but not limited to, diverse patient populations and therapeutic regimens) is still lacking, representing a critical barrier to routine clinical implementation. Only through such rigorous evaluation, organoid-guided therapeutic strategies could translate into routine clinical use.

Overall, the future of CRC organoid research lies in its ability to integrate organoid models, mechanistic studies, and predictive intelligence into a unified, closed-loop system that bridges the gap between experimental modeling and personalized medicine. Achieving this vision will require coordinated progress in bioengineering, computational biology, and clinical validation, but it also offers an unprecedented opportunity to redefine how we study and treat CRC.

## Data Availability

No new data were created or analyzed in this study.
